# Selection of RNA aptamers targeting hypoxia in cancer

**DOI:** 10.3389/fmolb.2022.956935

**Published:** 2022-09-14

**Authors:** Silvia Nuzzo, Margherita Iaboni, Maria Luigia Ibba, Anna Rienzo, Domenica Musumeci, Monica Franzese, Giuseppina Roscigno, Alessandra Affinito, Gianluca Petrillo, Cristina Quintavalle, Giuseppe Ciccone, Carla Lucia Esposito, Silvia Catuogno

**Affiliations:** ^1^ IRCCS SYNLAB SDN, Naples, Italy; ^2^ Bracco Imaging S.p.A., Turin, Italy; ^3^ Institute Experimental Endocrinology and Oncology “Gaetano Salvatore” (IEOS), National Research Council (CNR), Naples, Italy; ^4^ Department of Chemical Sciences, “Federico II” University of Naples, Naples, Italy; ^5^ Department of Molecular Medicine and Medical Biotechnology, “Federico II” University of Naples, Naples, Italy; ^6^ Percuros B.V., Enschede, Netherlands

**Keywords:** hypoxia, cell-SELEX, aptamer, cancer, detection

## Abstract

Hypoxia plays a crucial role in tumorigenesis and drug resistance, and it is recognised as a major factor affecting patient clinical outcome. Therefore, the detection of hypoxic areas within the tumour micro-environment represents a useful way to monitor tumour growth and patients’ responses to treatments, properly guiding the choice of the most suitable therapy. To date, non-invasive hypoxia imaging probes have been identified, but their applicability *in vivo* is strongly limited due to an inadequate resistance to the low oxygen concentration and the acidic pH of the tumour micro-environment. In this regard, nucleic acid aptamers represent very powerful tools thanks to their peculiar features, including high stability to harsh conditions and a small size, resulting in easy and efficient tumour penetration. Here, we describe a modified cell-SELEX (Systematic Evolution of Ligands by EXponential enrichment) approach that allows the isolation of specific RNA aptamers for the detection of the hypoxic phenotype in breast cancer (BC) cells. We demonstrated the effectiveness of the proposed method in isolating highly stable aptamers with an improved and specific binding to hypoxic cells. To our knowledge, this is the first example of a cell-SELEX approach properly designed and modified to select RNA aptamers against hypoxia-related epitopes expressed on tumour cell surfaces. The selected aptamers may provide new effective tools for targeting hypoxic areas within the tumour with great clinical potential.

## 1 Introduction

Hypoxia, generally referred to as a depressed tissue oxygen tension (∼5–10 mmHg *versus* 40–60 mmHg in normal conditions), is a common hallmark of most solid tumours, caused by the presence of an abnormal microvasculature in rapidly proliferating tissues. Tumour hypoxia can be of two types: 1) acute hypoxia, caused by a temporary defective vascular network in tumour tissue that reduces tumour perfusion and determines a short-term hypoxia (from a few minutes up to 72 h); 2) chronic hypoxia, determined by a high oxygen consumption by the cells close to vessels that leaves adequate oxygen quantities for distant cells (Bayer and Vaupel, 2012). In response to low oxygen levels, tumour cells develop an adaptive response, activating key regulators such as the hypoxia-inducible transcription factor (HIF) protein family that induce specific changes in gene expression and cell phenotype (Carmeliet et al., 1998).

It has been well established that hypoxia plays a key role in tumour progression, spreading and recurrence, with many reports identifying the hypoxic phenotype as a negative prognostic factor in cancer patients, being associated with an increased risk of treatment failure (chemotherapy and/or radiotherapy) and tumour metastasis development (Rofstad, 2000; Brown and Wilson, 2004; Vaupel and Mayer, 2007; Facciabene et al., 2011; Walsh et al., 2014; Muz et al., 2015; Horsman and Overgaard, 2016; Wigerup et al., 2016; Soleymani Abyaneh et al., 2018; Valencia-Cervantes et al., 2019). In addition, it has been demonstrated that the hypoxic niche in the tumour mass is enriched in cancer stem cells (CSC) and promotes their propagation, which is strongly associated with tumour recurrence (Najafi et al., 2020). Therefore, in order to improve the therapeutic efficacy and the patients’ clinical outcome, the selective detection of hypoxic cells while sparing normal tissues represents an important tool that can guide the choice towards the most suitable personalised therapeutic options for each patient.

The traditional method for hypoxia detection is the Clark electrode, an invasive method with a reduced sensitivity due to the difficulty of accessing the tumour mass (Kim et al., 1993; Calvo-Anguiano et al., 2018). Hence, in recent years, many efforts have been made in order to identify and develop new non-invasive methods for hypoxia detection, especially through appropriate imaging modalities, including positron emission tomography/computed tomography (PET/CT) (Minagawa et al., 2011), magnetic resonance imaging (MRI) (Hoskin et al., 2007), photoacoustic imaging (PAI) (Knox et al., 2018) and optical imaging (Zhang et al., 2009). However, the *in vivo* applicability for hypoxia detection of the imaging probes used in these methods is limited by their general non-optimal response to low oxygen levels, their lack of efficacy for all tumour types and their non-specific uptake (Hammond et al., 2014; Mirabello et al., 2018). Thus, the identification of new hypoxia-specific tools with improved properties could provide important advances in cancer targeting.

In this context, nucleic acid aptamers may represent a very interesting and powerful option. Aptamers are short single-stranded DNAs or RNAs that have emerged as useful molecules for both diagnostic and therapeutic scopes. Aptamers, assuming particular three-dimensional (3D) shapes, bind with high affinity and specificity to a given target and are often endowed with protein inhibitory activity (Fu and Xiang, 2020). Moreover, aptamers against cell surface receptors represent very attractive tools for the targeted delivery of a wide range of molecules. Indeed, upon binding to target cells, aptamers may undergo intracellular receptor-mediated uptake and can drive the specific internalization of secondary reagents for both diagnostic and therapeutic purposes (Zhou and Rossi, 2017; Guan and Zhang, 2020). Importantly, aptamers show many useful advantages for biomedical applications, including low immunogenicity, small size, high tissue penetration, and high plasticity, which render them suitable for different chemical modifications in order to improve their stability in harsh conditions, their pharmacokinetic and pharmacodynamic properties, and to properly modify them for various clinical uses (Zhang et al., 2019). Thanks to these properties, aptamers have already been explored as probes for optical imaging, PET/CT, and MRI in different tumour types (Guan and Zhang, 2020). Thus, they may represent ideal molecules for the development of non-invasive and more effective methods for hypoxia detection in the tumour site.

Aptamers are selected *in vitro* by the SELEX (Systematic Evolution of Ligands by EXponential enrichment) technology. Such an approach has been successfully applied to different targets, ranging from small molecules to complex systems, such as whole cells. The last strategy, named whole cell-SELEX, allows the isolation of aptamers against a specific cell phenotype and binding of cell membrane proteins in their native conformation (Catuogno and Esposito, 2017).

In order to isolate aptamers able to specifically recognize hypoxia-related cell surface epitopes, we designed a cell-SELEX protocol properly modified for this purpose and referred to as “Hypoxic cell-SELEX”. We used Triple Negative Breast Cancer (TNBC) cells chemically induced with Cobalt (II) Chloride hexahydrate (CoCl_2_) as the target in the *“positive selection step”.* This type of approach has the advantage of using a simple and rapid system to reproduce *in vitro* the hypoxic conditions present in the tumour micro-environment and allows the selection of aptamers specific to this particular cellular phenotype using a non-laborious procedure, not much more complex than the classic cell-SELEX approach.

By such a strategy, we identified a pool of aptamers with improved binding activity on the hypoxic phenotype, also when it is induced by more physiological systems, such as the hypoxia incubator chamber. The identification of one of the most represented aptamers with improved binding affinity for the hypoxic phenotype, as well as its biophysical characterization, are here reported in view of specifically targeting hypoxic areas within the tumour mass.

## 2 Materials and methods

### 2.1 Cell culture and hypoxia induction

The MDA-MB-231 cell line, purchased from the ATCC (LG Standards, Milan, Italy), was grown in Dulbecco’s modified Eagle’s medium (DMEM) supplemented with 10% heat-inactivated fetal bovine serum (FBS) and 100 U/ml penicillin/streptomycin. All cell culture reagents were purchased from Sigma (St Louis, MO, United States). Primary cultures, Pt.170 (TNBC) and Pt.160 (Luminal B-HER2^−^), were grown in DMEM-F12 (Sigma) supplemented with 10% FBS and 100 U/ml penicillin/streptomycin.

Hypoxia induction by Chemical method: 2 × 10^5^ MDA-MB-231 cells were seeded in p35 cell plate and incubated with 100, 150, 200 μM of Cobalt (II) Chloride hexahydrate (Sigma, St Louis, MO, United States) in a conventional incubator (37°C; 5% CO_2_). Cobalt (II) Chloride hexahydrate was dissolved in serum-free medium before use. Hypoxia induction by physical method: 2 × 10^5^ MDA-MB-231 cells were seeded in a p35 cell plate in serum-free medium and put in the hypoxic incubation chamber maintained at 5% O_2_. The cells were recovered at 24 h.

### 2.2 Immunoblotting

Cells were washed twice in ice-cold phosphate-buffered saline (PBS) (Sigma St Louis, MO, United States) and lysed in the JS buffer containing: 50 mM Tris-HCl pH 7.5, 150 mM NaCl, 1% Nonidet P-40, 2 mg/ml aprotinin, 1 mg/ml pepstatin, 2 mg/ml leupeptin, 10 mM Na_3_VO_4_. Protein concentration was determined by Bradford assay (Biorad, Hercules, CA, United States). Cell lysates were denatured in Laemmli buffer (2% SDS, 5% β-mercaptoethanol, 0.001% Bromophenol Blue, 10% glycerol) for 5 min at 100°C and then subjected to SDS-PAGE. 12% Acrylamide/bis-acrylamide gels were electroblotted into polyvinylidene difluoride (PVDF) membranes (Millipore Co., Bedford, MA, United States). Membranes were then probed with primary antibodies as indicated. The primary antibodies used were: anti-CA-IX (R&D Systems, Minneapolis, MN. United States); anti-HIF-1α (BD Biosciences, San Jose, United States); anti-α-tubulin (Santa Cruz Biotechnology, CA, United States); anti-β-actin (Santa Cruz Biotechnology, CA, United States). Donkey anti-goat, goat anti-mouse, and goat anti-rabbit (Santa Cruz Biotechnology, CA, United States) were used as secondary antibodies. Band intensity was measured with the Image J program.

### 2.3 Immunofluorescence

To assess CA-IX expression on the cell surface, MDA-MB-231 cells in normoxic conditions or stimulated with CoCl_2_ (150 μM) for 24 h to mimic hypoxic conditions were seeded on poly-L-Lysine coated glass cover slips and incubated with anti-CA-IX antibody for 30 min at 37°C prior to fixation. Cells were then fixed with paraformaldehyde 4% in PBS for 10 min and incubated with Alexa-488 secondary antibody (Invitrogen, Waltham, MA, United States) at 37°C for 30 min. Cover slips were then mounted on microscope slides with Prolong Gold Antifade Reagent with DAPI (Invitrogen, Waltham, MA, United States) and visualised by confocal microscopy. Images were obtained using a Zeiss 510 LSM confocal microscope with a 40× oil objective. For each image, three background areas were used to normalize against auto-fluorescence. Fluorescence intensity was quantified with the Image J program, excluding the nucleus signals and measuring the area, integrated density, and mean grey value of each cell/image as described (Jakic et al., 2017). Corrected total cell fluorescence (CTCF) was measured according to the formula CTCF = integrated density—(area of selected cell × mean fluorescence of background readings), considering four background areas to normalize for each image.

### 2.4 Hypoxic cell-systematic evolution of ligands by exponential enrichment method

The SELEX cycle was performed with an *in vitro* transcribed RNA pool. Transcription was performed in the presence of 1 mM 2′-F pyrimidines (2′-F-Py) and mutant T7 RNA polymerase (2.5 U/mL T7 R&DNA polymerase, Epicentre Biotechnologies, Madison, Wisconsin) to improve yields. 2′F-Py-nbucleotides were used to increase the resistance of the aptamers to degradation by serum nucleases. Before incubation, 2′F-Py RNAs were heated at 85°C for 5 min, snap-cooled on ice for 2 min, and allowed to warm up to 37°C. The Hypoxic cell-SELEX protocol is composed of twelve cycles.

#### 2.4.1 Counter-selection step against normoxic MDA-MB-231 cells

To avoid the selection of aptamers recognizing normoxic targets on MDA- MB-231 surface, the pool was first incubated on normoxic MDA-MB-231 for 30 min (up to round 6) or for 15 min (for the following rounds) at 37°C. In each cycle, the SELEX conditions were changed, such as the dimension of the cell culture dishes (in sequence: 150, 100, 50, 35 mm). Unbound sequences were recovered for the selection step.

#### 2.4.2 Selection step against hypoxic MDA-MB-231cells

The recovered sequences were incubated on CoCl_2_-stimulated MDA-MB-231 cells (to mimic the hypoxic condition). After several washes, sequences were recovered by total RNA extraction.

During the selection process, the selective pressure was changed, increasing the washing number (from one for the first cycle up to five for the last cycles), decreasing the incubation time (from 30 to 15 min from round 7) and the dimension of the cell culture dishes (in sequence 150, 100, 50, 35 mm). It also increased the number of counter-selection steps from one to two from round 8. Moreover, the use of a non-specific competitor, named yeast tRNA (Sigma, St Louis, MO, United States), was introduced at different concentrations: 100 μg/ml for the round 10 and 200 μg/ml for the round 11; in the last two cycles (11 and 12) pre-treatment with yeast tRNA before of the 2′F-Py RNAs pool incubation was made.

### 2.5 Reverse transcription-polymerase chain reaction, mutagenic and non-mutagenic polymerase chain reactions for the cell-systematic evolution of ligands by exponential enrichment method

The RNA extracted from each SELEX cycle was retro-transcribed using Tetro Reverse Transcriptase Enzyme (Bioline, London, United Kingdom) according to the manufacturer’s protocol. The retro-transcription reaction was as follows: 90°C for 3 min, 42°C for 15 min and 50°C for 30 min. The product was used for a mutagenic PCR, characterised by a higher concentration of MgCl_2_ (6 mM) and dNTP (1 mM), using 0.5 U/μL of FIREpol DNA Polymerase (Microtech, Milan, Italy) and 0.3 μM primers:

N40 (Forward): 5′- TTC​AGG​TAA​TAC​GAC​TCA​CTA​TAG​GGA​AGA​GAA​GGA​CAT​ATG​AT-3′

N40 (Reverse): 5′-TCA​AGT​GGT​CAT​GTA​CTA​GTC​AA -3′

The PCR reaction was as follows: 95°C for 5 min, 10 cycles of 95°C for 1 min, 65°C for 1 min and 72°C for 1 min, and a final extension of 72°C for 1 min. The last SELEX cycle was amplified by a non-mutagenic PCR using 0.1 U/μL of FIREpol DNA Polymerase and 200 μM dNTP, without adding MgCl_2_ to that contained in the Taq Buffer. The reaction was as follows: 95°C for 5 min, 8 cycles of 95°C for 30 s, 65°C for 1 min, and 72°C for 1 min, and a final extension of 72°C for 5 min. Amplified DNA was purified using Amicon Ultra Centrifugal Filters (Millipore, MA, United States). After the last SELEX cycle, sequences from the pools were subjected to cloning with the TOPO-TA cloning kit (Invitrogen, Waltham, MA, United States). Sequences were analysed for multiple alignment (ClustalW by EMBL-EBI).

### 2.6 Cell binding assay

The binding assay of individual aptamers or aptamer pools was performed in 6-well plates on normoxic cells as negative cells and hypoxic cells as positive cells. For CoCl_2_-induced binding, cells (1.5 × 10^5^ per well) were seeded and treated with 150 µM CoCl_2_ in serum-free medium. After 24 h, cells were incubated for 30 min with serum-free medium in the presence of yeast tRNA 200 ng/μL. Then, 100 nM of RNA pools (H0, H12) or aptamer (MA 39/76) was added and incubated for an additional 30 min at 37°C. Following three washes with PBS to remove unbound aptamers, the bound sequences were recovered. Binding with hypoxic chamber-induced cells was performed by directly incubating 100 nM of H12 or the aptamer with cells for 15 min at 37°C. At the end of incubation, cells were immediately put at a cold temperature (on ice) to slow proteolytic degradation and then washed and lysed.

For affinity measurement, CoCl_2_-induced MDA-MB-231 cells were incubated for 30 min with yeast tRNA 200 ng/μL and then with increasing MA 39/76 concentrations (from 25 to 400 nM) for an additional 30 min.

Bound sequences were recovered by TRIzol (Life Technologies, Carlsbad, CA, United States) containing 0.5 pmol/ml of reference control. The amount of bound RNAs was determined by reverse transcription quantitative real-time PCR (RT-qPCR). At each experiment, the cells cultured were counted. The obtained data were normalised to the reference control and to cell number.

Affinity constant (K_D_) was calculated by plotting the data with GraphPad Prism Software, applying a non-linear curve fitting algorithm and the following equation: Y = Bmax * X/(K_D_ + X).

### 2.7 *In vitro* human serum stability

The MA 39/76 aptamer was incubated at 4 μM concentration in 80% human serum (Type AB Human Serum provided by Euroclone, Milan, Italy) for 168 h. At each time point (from 1 h to 168 h), 16 pmol of samples were recovered and incubated for 1 h at 37°C with 5 μL of proteinase K solution (600 mAU/mL) to minimize serum proteins that impair electrophoretic migration. Following the addition of 18 μL of RNA dye (Invitrogen, Waltham, MA, United States), samples were kept at −80°C until subsequent analysis. All time point samples were loaded into a 15% polyacrylamide/urea 7 M denaturing gel and separated by electrophoresis. The gel was stained with ethidium bromide and exposed to UV light.

### 2.8 *In vitro* pH stability by gel electrophoresis

Phosphate-buffered saline (PBS) solutions were prepared at different pHs (from 2 to 9) and boiled for 30 min at 100°C to inactivate RNAse. The H12 pool or MA 39/76 (8 pmol) was incubated at 37°C with all the solutions. After 3 h, all the samples were recovered and 2x RNA dye (Invitrogen, Waltham, MA, United States) was added to each sample. All samples were loaded into a 15% polyacrylamide/urea 7 M denaturing gel and separated by electrophoresis. The gel was stained with ethidium bromide and exposed to UV light.

### 2.9 Circular dichroism and UV spectroscopy

Circular dichroism (CD) spectra were performed in a quartz cuvette with a path length of 1 cm (3 ml internal volume, Hellma) on a Jasco J-1500 spectropolarimeter equipped with a Jasco CTU-100 circulating thermostat unit. Spectra were recorded at 20 and 37°C in the 220–320 nm range with a 2 s response, a 100 nm/min scanning speed, and 2.0 nm bandwidth, corrected by subtraction of the background scan (buffer) and averaged over 3 scans. CD analysis experiments were carried out on aptamer samples in PBS buffer at the indicated pH and at a 0.5 μM concentration. The aptamer (MA 39/76) was diluted from a stock concentrated solution in nuclease-free water to PBS at the indicated pH at 0.5 μM concentration, and CD analysis of each solution was performed immediately after dilution (t = 0), after 3 and 48 h incubation at 37°C. The PBS buffer was adjusted at three different pH (5.0, 7.0, and 9.0) and boiled for 30 min at 100°C to inactivate RNAses before diluting the aptamer.

The UV spectra were obtained on a Jasco V-770 UV-Visible/NIR spectrophotometer equipped with the ETCS-761 Peltier thermostatted single cell holder, using a 0.5 cm path length cuvette (1.5 ml internal volume, Hellma). Spectra were recorded in the 200–320 nm range using a scanning speed of 100 nm/min and a 2.0 nm bandwidth with appropriate baseline subtraction. The UV spectra and the UV-melting experiment were carried out on the aptamer sample at 0.5 μM concentration, after its incubation for 3 h in the PBS buffer at pH 5. The UV-melting was recorded by monitoring the absorbance at 260 nm upon increasing the temperature at 1 °C/min.

### 2.10 Statistics

Statistical analysis was performed using GraphPad Prism Software. Student’s *t*-test was applied for comparison between the two groups. For statistical significance we considered a *p* value < 0.05.

## 3 Results and discussion

### 3.1 Hypoxia induction in MDA-MB-231 cells

We aimed to develop a selection procedure to isolate aptamers able to specifically recognize hypoxia-related epitopes on tumour cell surfaces. To this end, as a first attempt, we identified the more adequate conditions to select aptamers targeting hypoxia-related cell surface epitopes. The hypoxia phenotype can be induced *in vitro* by both physical and chemical methods. The physical method is represented by the hypoxia incubator chamber that maintains low O_2_ levels (1%–5%), while the chemical methods exploit various molecules, such as CoCl_2_, dimethyloxalylglycine (DMOG), desferrioxamin (DFO), and MG132, to induce changes in gene expression, mimicking those occurring under hypoxic conditions (Wu and Yotnda, 2011). Among them, CoCl_2_ is the most frequently used (Munoz-Sanchez and Chanez-Cardenas, 2019). It mimics hypoxia by inhibiting the prolyl-hydroxylase domain-containing proteins (PHDs), which regulate HIF-1α degradation through its hydroxylation within the oxygen-dependent degradation domain (ODD), and the blocking of the factor inhibiting HIF-1 (FIH), responsible for the repression of the HIF-1α-induced pathways. Consequently, the von Hippel-Lindau protein (pVHL), part of the E3 ubiquitin ligase complex responsible for HIF-1α proteasomal degradation, cannot bind the hydroxylated ODD to induce protein degradation. Thus, HIF-1α is stabilised and translocated to the nucleus where it activates hypoxia-responsive genes.

**FIGURE 1:**
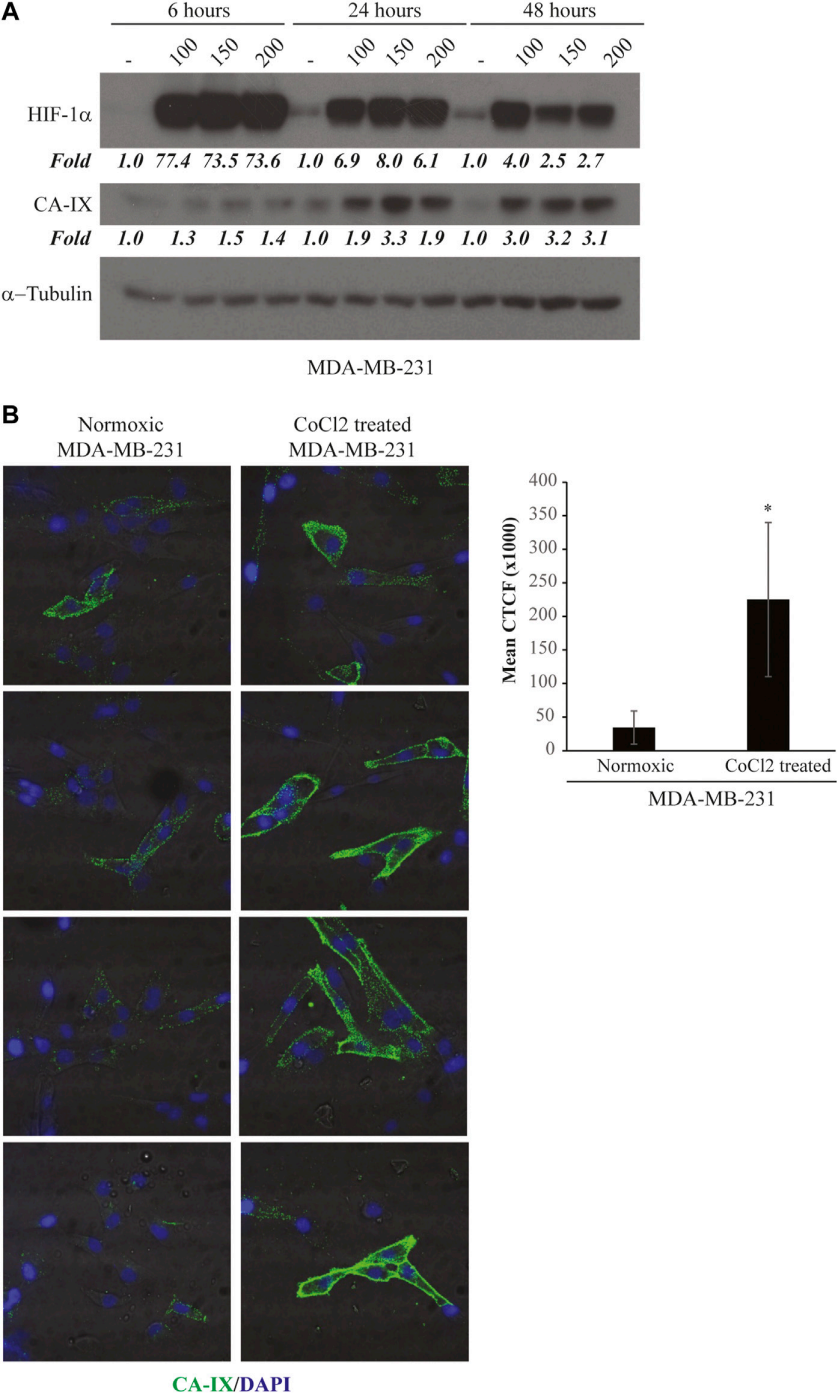
CoCl2-induced hypoxic phenotype. **(A)** Time and dose-response assay for CA-IX and HIF-1α expression. MDA-MB-231 cells were treated with increasing concentrations of CoCl_2_ (100–150–200 μM) for 6, 24 or 48 h. Cell lysates were recovered and immunoblot analysis was performed to evaluate CA-IX and HIF-1α expression; β-actin was used to monitor equal loading. Values below the blot indicate the band intensity quantified by the Image J program. Signals have been normalised over the loading control and expressed relative to the correspondent untreated point. **(B)** CA-IX expression on MDA-MB-231 cell surface. MDA-MB-231 cells in normoxic conditions or treated with 150 μM of CoCl_2_ for 24 h were incubated with CA-IX primary antibody and fixed. The Alexa-488-labeled secondary antibody was used to detect the fluorescence signal by confocal microscopy. *Left panels*, the image shows four representative fields of the same slide. All images were captured with the same settings, enabling direct comparison of staining patterns. *Right panel*, Florescence intensity of confocal microscopy images (*n* = 4) was quantified by the Image J program and expressed as mean Corrected Total Cell Fluorescence (CTCF). The error bars indicate the standard deviation. Statistics by Student’s t-test are indicated: *, *p* < 0.05.

The use of the hypoxia incubator chamber is the most physiological way reproduce the hypoxic phenotype *in vitro*. However, there are difficulties in maintaining the hypoxic conditions constant due to the inevitable entry of oxygen at each opening of the chamber. Therefore, the use of CoCl_2_, which has demonstrated comparable and efficient induction of hypoxia-related gene expression (Calvo-Anguiano et al., 2018), is preferable, especially when multi-step experimental procedures, such as cell-SELEX, are required. For these reasons, for the hypoxic cell-SELEX strategy, we decided to induce hypoxia through CoCl_2_ treatment. As target cells, we used MDA-MB-231 cells, a model of TNBC, the most aggressive and unsolved form of BC for which the prognostic and pro-tumour role of hypoxia has been widely demonstrated (Dong et al., 2013; Nie et al., 2018; Cui and Jiang, 2019; Zhao et al., 2020). In order to set the CoCl_2_ conditions to use in the SELEX procedure, we treated MDA-MB-231 cells with different concentrations of CoCl_2_ for different times and monitored the hypoxia induction by analysing the levels of HIF-1α and its dependent target, the carbonic anhydrase IX (CA-IX) (Figure 1A). In agreement with previous reports (Li et al., 2009), we observed that in MDA-MB-231 cells, CoCl_2_ increased HIF-1α levels before those of CA-IX. In fact, HIF-1α up-regulation was strongly visible 6 h after treatment. Instead, levels of CA-IX well increase following 24 h treatment (about 3-fold of increase over control), showing the higher induction at 150 μM of CoCl_2_ concentration (Figure 1A).

Next, in order to confirm the induction of a hypoxic cell surface phenotype, we checked if the hypoxic marker CA-IX was expressed in the right localization, i.e., on the cell membrane surface. To this end, we monitored CA-IX levels by immunofluorescence at the optimal CoCl_2_ concentration and treatment time identified. As shown (Figure 1B), CA-IX was significantly up-regulated upon CoCl_2_ treatment, as compared with untreated cells, and mainly localised on the cell surface.

These data indicate that cell treatment with CoCl_2_ at a concentration of 150 μM for 24 h is an optimal condition to induce a hypoxic-like phenotype with an efficient over-expression of specific cell surface hypoxic markers. Therefore, this condition was chosen for the “Hypoxic cell-SELEX” procedure.

### 3.2 Selection of aptamers binding to hypoxic MDA-MB-231 cells

In order to select specific aptamers for hypoxia-related epitopes, we designed a suitably modified protocol, called “Hypoxic cell-SELEX”. We modified the classic cell-SELEX strategy, which can be applied for targeting a specific cell phenotype and allows the selection of aptamers in a physiological context, thus improving their performance *in vivo*.

**FIGURE 2:**
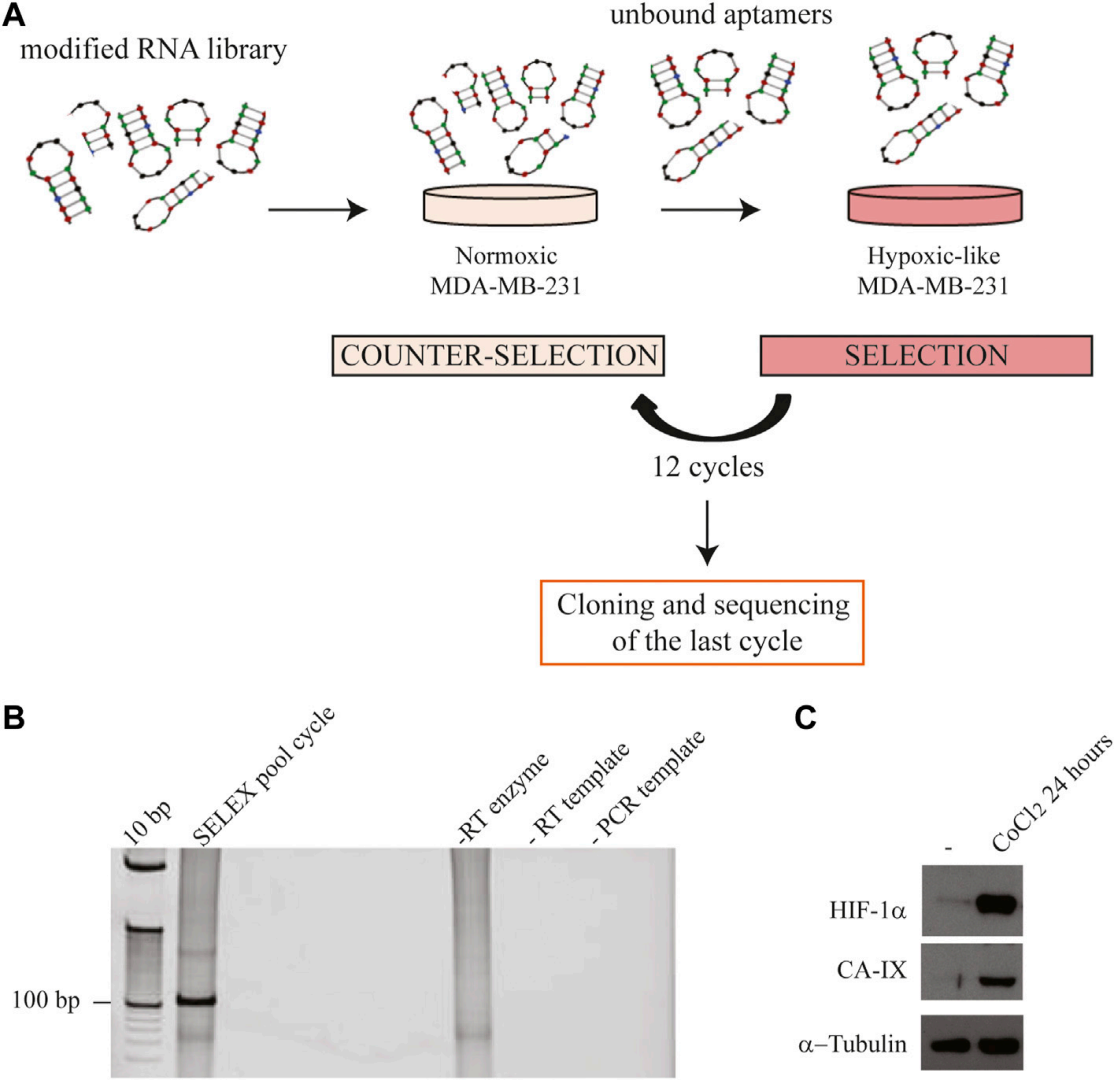
Hypoxic cell-SELEX strategy. **(A)** Each SELEX round included one or two “*counter-selection steps”* on normoxic MDA-MB-231 and one “*selection step”* on CoCl_2_-induced MDA-MB-231 cells (hypoxic-like). **(B)** Representative RT-PCR amplification of the aptamer pool was obtained following a SELEX round. Samples were loaded onto a 12% polyacrylamide gel, stained with ethidium bromide and visualised by UV exposure. Control samples: “-RT enzyme” indicates the pool reverse-transcribed without the reverse-transcriptase enzyme, performed to exclude DNA contamination in RT; “-RT template” indicates the amplification of the product from an RT reaction performed without the RNA template, to exclude RNA contamination in RT; “- PCR template” indicates the negative control of the PCR reaction; **(C)** Representative immunoblot analysis of HIF-1α and CA-IX expression on normoxic (used in the “*counter-selection step”*) and CoCl_2_-induced hypoxic MDA-MB-231 cells (used in the “*selection step”*). α-Tubulin was used as a housekeeping reference.

As a starting aptamer pool, we used a high complexity library consisting of 2′-F-Py RNAs with improved resistance to serum nucleases. To favour the selection for aptamers able to efficiently discriminate the normoxic from the hypoxic phenotype, at each round, the “*selection step”* on hypoxic MDA-MB-231 cells induced with CoCl_2_ was preceded by one or two “*counter-selection steps”* on parental MDA-MB-231 in normoxic conditions (Figure 2A). We performed 12 rounds, progressively increasing the selective pressure through the procedure by: 1) reducing the cell number and the incubation time; 2) increasing the washing number; and 3) adding an excess of poly anionic competitor (yeast tRNA) to remove unspecific binders (see Table 1).

**TABLE 1:** Hypoxic cell-SELEX conditions.

Round	Cell confluence (80%)	RNA amount (μg/ml)	Incubation time (min)	Wash number	Counter-selection number	Yeast tRNA (μg/ml)	Preincubation with yeast tRNA (μg/ml)
1	p150	1.2	30	1	1	—	—
2	p150	1.2	30	2	1	—	—
3	p150	1.2	30	3	1	—	—
4	p100	1.8	30	4	1	—	—
5	p100	1.8	30	4	1	—	—
6	p100	1.8	30	5	1	—	—
7	p100	1.8	15	5	1	—	—
8	p100	1.8	15	5	2	—	—
9	p60	3.9	15	5	2	—	—
10	p35	9	15	5	2	100	—
11	p35	9	15	6	2	100	100
12	p35	9	15	6	2	200	200

At each SELEX round, the aptamer library bound to hypoxic MDA-MB-231 cells was subjected to RT-PCR and the obtained product was checked on a polyacrylamide gel (Figure 2B). The expression of HIF-1α and CA-IX proteins on target cells was also evaluated by immunoblot to monitor the efficient induction of the hypoxic-like phenotype (Figure 2C).

Generally, 10–15 rounds of cell-SELEX are sufficient to isolate aptamers against the desired target and to avoid the loss of promising sequences recognizing less enriched epitopes or the reduction of the enrichment due to too extensive mutagenesis (Komarova and Kuznetsov, 2019; Kohlberger and Gadermaier, 2021). Therefore, we stopped the selection procedure at round 12 in order to maintain a good balance between the binding enrichment and the recovery rate of sequences of potential interest. At the end of round 12, the binding capacity of the pool was properly analysed to confirm the enrichment for hypoxia-specific aptamers.

**FIGURE 3:**
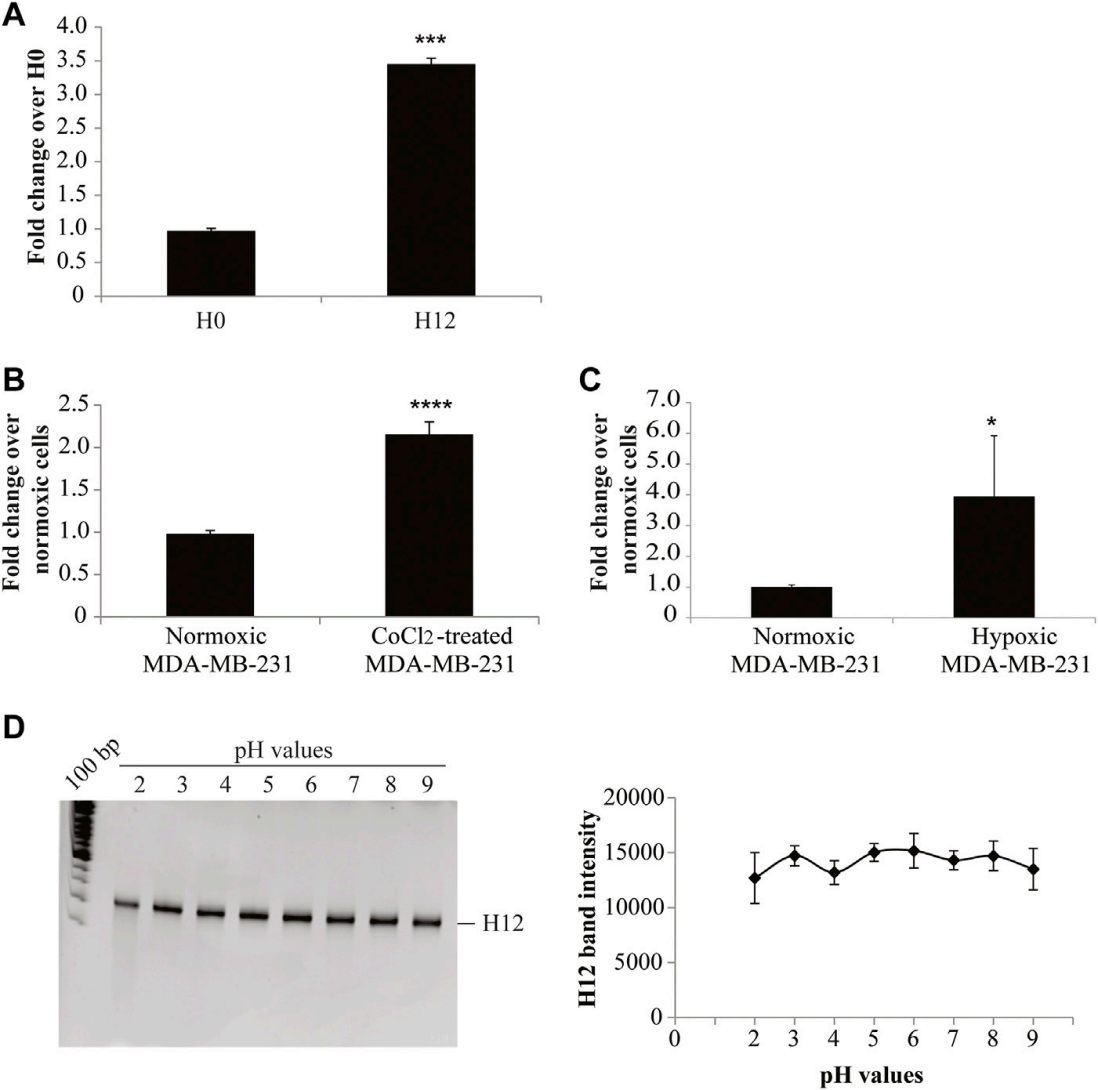
H12 characterization. **(A)** The binding capability of the starting and the final aptamer pool (indicated as H0 and H12, respectively) on CoCl_2_-induced MDA-MB-231 cells was analysed by an RT-qPCR assay. In the graph are indicated the fold changes over H0. The error bars show the mean ± standard deviation values on duplicates (*n* = 2) and statistics were calculated using Student’s t-test, ***p < 0.001; **(B)** Binding assay of H12 by RT-qPCR performed on CoCl_2_-induced and normoxic MDA-MB-231 cells, used as a negative control. In the graph we indicate the fold changes calculated over normoxic MDA-MB-231 cells. Error bars show the mean ± standard deviation values on replicates (*n* = 4) and statistics were calculated using Student’s t-test, ****p < 0.0001. **(C)** Binding assay of H12 by RT-qPCR performed on hypoxic chamber-mediate MDA-MB-231 and normoxic MDA-MB-231 cells, used as a negative control. In the graph we indicated the fold changes calculated over normoxic MDA-MB-231 cells. The error bars show the mean ± standard deviation values on replicates (*n* = 4), and statistics were calculated using Student’s t-test, *p < 0.05. **(D)** pH stability of the H12 pool was measured by incubating the pool in buffers with different pH (2, 3, 4, 5, 6, 7, 8, 9) for 3 h at 37°C. RNA sequences were recovered and subjected to a 15% denaturing polyacrylamide gel (*left panel*). Lane 1: 100 bp DNA ladder. After the staining with ethidium bromide, the gel was visualised on the Gel Doc EZ Imager and bands were quantified by using the Image J program. The pixel intensity of the band for each pH value is reported in the graph (*right panel*). The error bars show the mean ± standard deviation values replicated.

Firstly, we checked the binding on hypoxic CoCl_2_-induced MDA-MB-231 cells. For this purpose, the starting (H0) and the final enriched aptamer pool (H12) were incubated with hypoxic MDA-MB-231 target cells and an RT-qPCR-based binding assay was performed. As shown in Figure 3A, the final pool (H12) presented an increased binding efficacy on hypoxic MDA-MB-231 target cells compared to the starting pool (H0), showing a fold-increase of about 3. Most importantly, the obtained final aptamer pool revealed to effectively discriminate CoCl_2_-induced MDA-MB-231 cells from parental MDA-MB-231 cells grown in normoxic conditions (Figure 3B). As previously reported, CoCl_2_ is only one of the ways used to reproduce hypoxia *in vitro*. In particular, the hypoxic chamber is the approach of choice to physiologically mimic the hypoxia conditions. For this reason, we also tested the final aptamer pool (H12) on hypoxic chamber-induced MDA-MB-231. Given that some hypoxia-related proteins may have a short half-life upon oxygen reintroduction (Jewell et al., 2001), the binding protocol was optimised (see methods for details) to limit the possibility of losing hypoxia-related markers. Notably, as shown in Figure 3C, H12 pool was able to discriminate between normoxic MDA-MB-231 from those grown in the hypoxic chamber, confirming the pool’s ability to recognize the hypoxic phenotype regardless of the technique employed to induce hypoxia.

Collectively, these data indicate that 12 rounds of SELEX were effective in isolating an aptamer pool enriched in sequences with improved specificity for the hypoxic phenotype.

A key aspect of an ideal hypoxia tracer is the ability to remain stable in regions with an acidic pH (Lopci et al., 2014; Fleming et al., 2015). We, thus, analysed the resistance to pH changes of the pool obtained from SELEX by incubating H12 in buffers with different pH (from 2 to 9). The stability of the oligonucleotides was checked by gel electrophoresis in denaturing conditions. The H12 pool was demonstrated to be stable in both acidic and basic pH conditions (Figure 3D).

Altogether, our data indicate that the developed SELEX procedure allows the isolation of a pool of aptamers specific for hypoxic cells, which may serve as probes for hypoxia detection in the tumour mass.

### 3.3 Identification of individual aptamers

In order to isolate individual aptamers, the final pool from SELEX was then subjected to sequencing. Although Next Generation Sequencing (NGS) can provide a broader analysis of the enriched aptamers and some information about pool evolution during the selection process, the Sanger sequencing method still represents a simple and cost-effective way to have a representative picture of the final aptamer pool, obtaining a sufficient and reasonable number of candidate sequences for further characterization. In addition, some studies using both Sanger sequencing and NGS revealed that the same winning sequences can be identified with both techniques (Stoltenburg and Strehlitz, 2018; Affinito et al., 2019).

**FIGURE 4:**
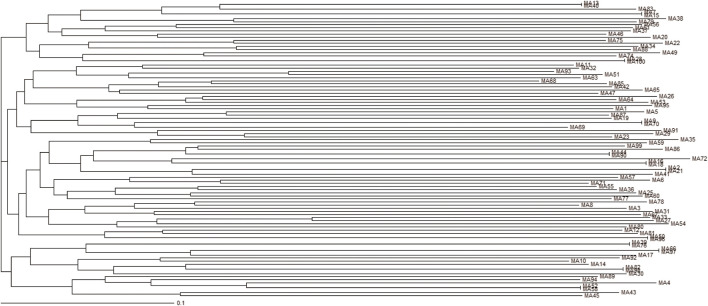
Phylogenetic dendrogram. Multiple Alignment of aptamers identified following cloning of the last hypoxic cell-SELEX round and sequencing. Dendrogram using Clustal Omega Multiple Sequence Alignment tool by EMBL-EBI.

Based on these considerations, the final H12 pool was cloned, and one hundred individual clones were sequenced (Supplementary Table S1). Individual sequences were regrouped into quasi-phylogenetic families based on their primary sequence similarities. As shown in Figure 4, in the phylogenetic dendrogram emerged twelve aptamer sequences repeated two times (MA 13/40, 7/-15, 16/-18, 2/-21, 39/-76, 50/-96, 52/-58, 9/-70, 98/-82, 44/-90, 66/-97, and 28/-100), covering 24% of all individual sequences obtained from the sequencing.

**FIGURE 5:**
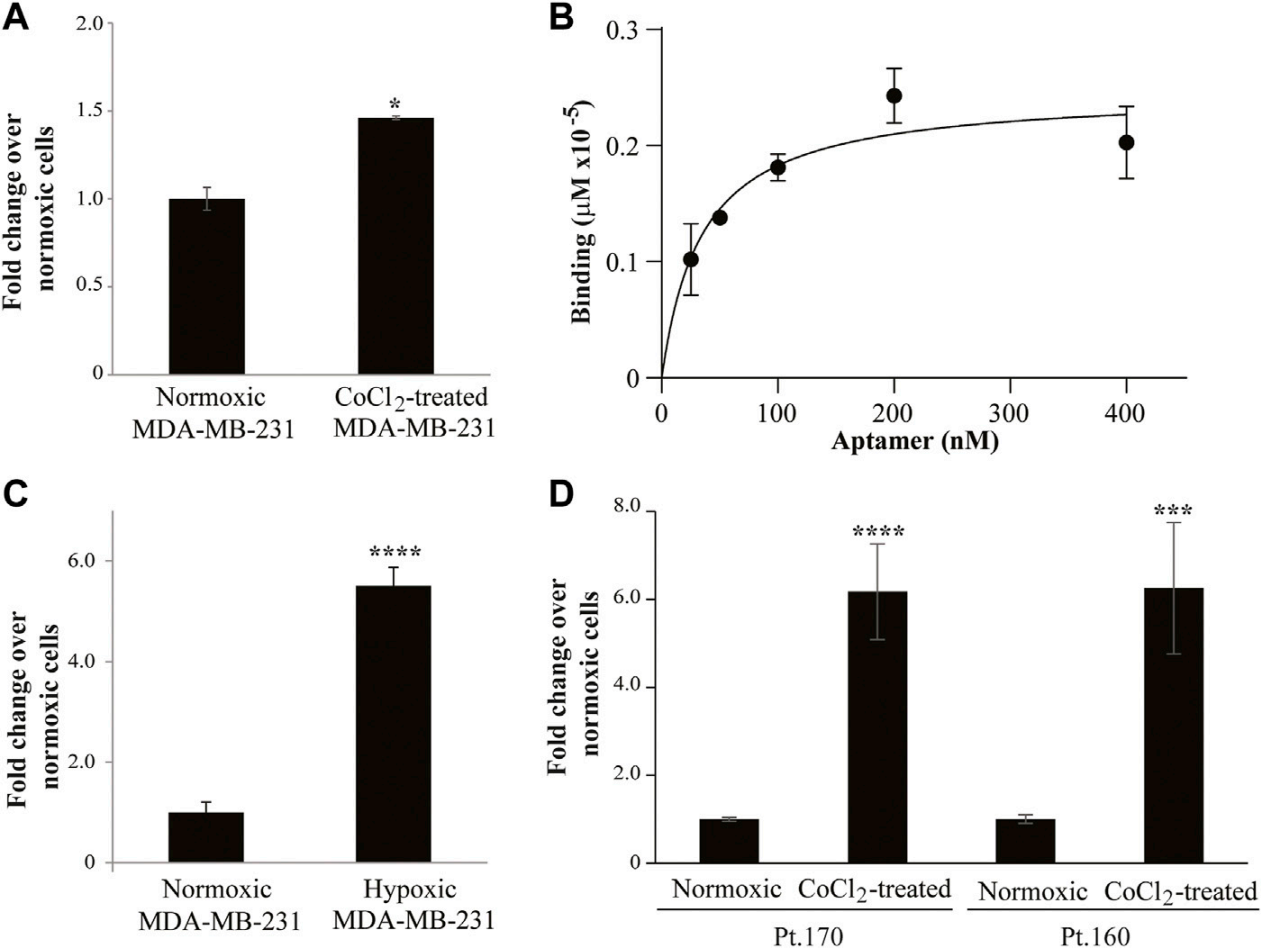
MA 39/76 aptamer binding ability. **(A)** MA 39/76 (100 nM) was incubated on CoCl_2_-induced MDA-MB-231 hypoxic cells or normoxic MDA-MB-231 cells for 30 min at 37°C. Binding values were measured by qRT-PCR. The error bars show the mean ± standard deviation values on duplicates (*n* = 2) and statistics by Student’s t-test were performed, *p < 0.05. **(B)** Dose-dependent binding of MA 39/76 on CoCl_2_-induced MDA-MB-231 hypoxic cells. Error bars show the mean ± standard deviation. **(C)** MA 39/76 100 nM was incubated with hypoxic chamber-mediated MDA-MB-231 cells or normoxic MDA-MB-231 for 15 min at 37°C. Binding values were measured by qRT-PCR. **(D)** Binding of 100 nM MA 39/76 on normoxic or CoCl_2_-induced primary BC cells (Pt.170 and Pt.160). In **(C,D)** Mean ± standard deviation values on replicates (*n* = 4) are reported; statistics by Student’s t-test, ***, *p* < 0.001****p < 0.0001.

As a proof of concept of the effective isolation of hypoxia-specific aptamers, we tested the binding specificity of one of the enriched sequences, MA 39/76. The binding was evaluated on hypoxic CoCl_2_-induced MDA-MB-231 cells. The MA 39/76 was able to efficiently bind the hypoxic phenotype (Figure 5A) and showed an estimated dissociation constant (K_D_) of ∼35 nM (Figure 5B), in line with affinity values of aptamers identified by cell-SELEX (Darmostuk et al., 2015; Camorani et al., 2022). In addition, the aptamer effectively discriminated hypoxic chamber-mediated MDA-MB-231 cells as well (Figure 5C). Importantly, the ability of MA 39/76 to recognize hypoxia-related epitopes was confirmed on two patient-derived BC cells (Figure 5D).

**FIGURE 6:**
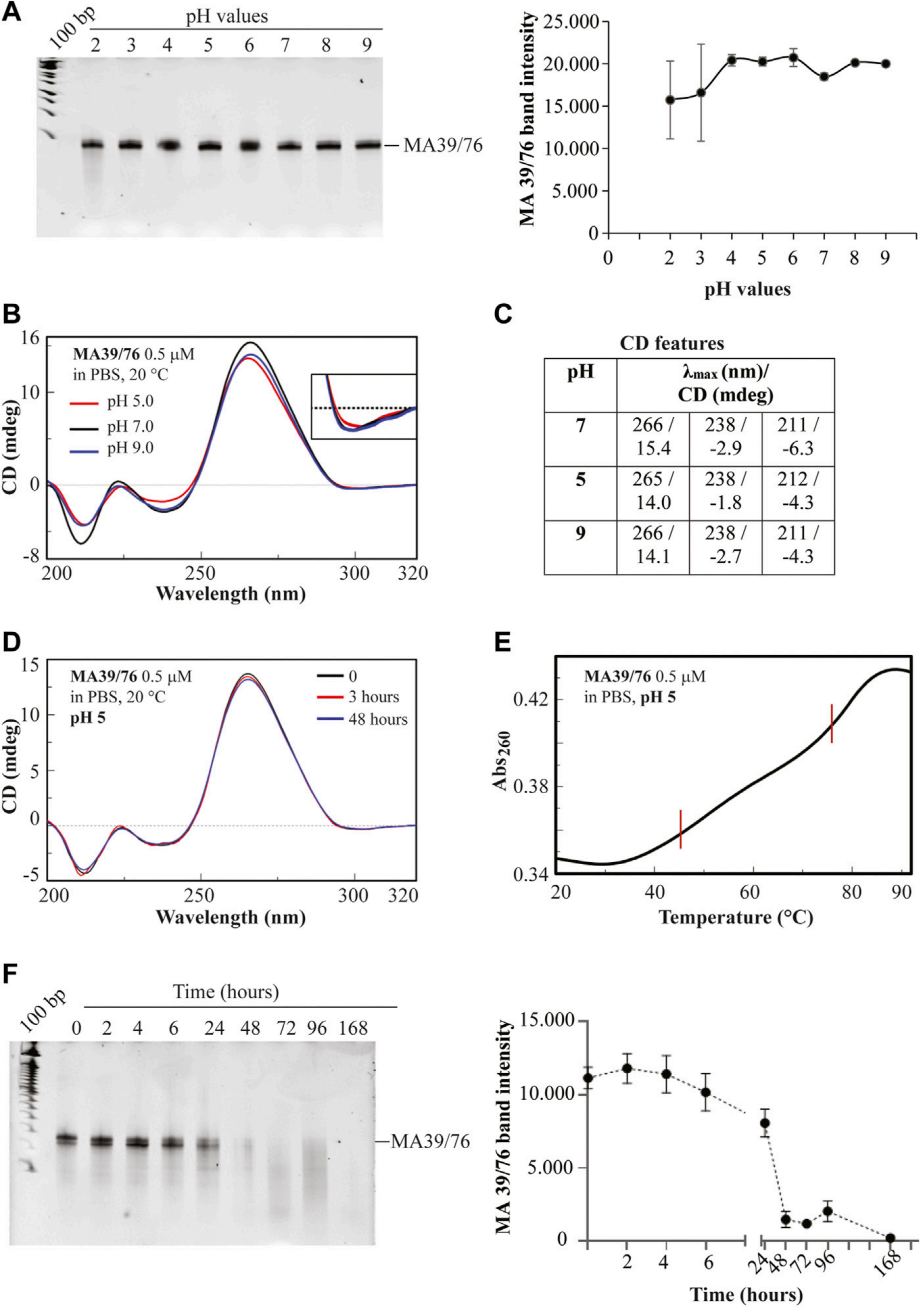
MA 39/76 aptamer stability. **(A)** pH stability of MA 39/76 was measured by incubating the aptamer in buffers with different pH (2, 3, 4, 5, 6, 7, 8, 9). RNA sequences were recovered and subjected to a 15% denaturing polyacrylamide gel (*left panel*). After staining, bands were quantified by using the ImageJ program. The pixel of band intensity for each pH value is reported in the graph (*right panel*). **(B)** Overlapped CD spectra of MA 39/76 aptamer at 0.5 μM concentration in PBS buffer at the indicated pH and **(C)** corresponding CD spectral features; **(D)** Overlapped CD spectra of the aptamer at 0.5 μM concentration recorded (20°C, 1 cm quartz cuvette) immediately after dilution in PBS buffer at pH 5 (time = 0), and after 3 and 48 h incubation at 37°C. **(E)** UV melting curve of the MA 36/76 aptamer at 0.5 μM concentration recorded in PBS buffer at pH 5 monitoring the absorbance at 260 nm upon increasing the temperature from 20 to 92°C (1°C/min; 0.5 cm quartz cuvette). **(F)** MA 39/76 serum stability was measured by incubating the aptamer at 4 μM in 80% human serum for 0, 2, 4, 6, 24, 48, 72, 96, 168 h. RNA sequences recovered were subjected to a 15% denaturing polyacrylamide gel. After the staining, bands were quantified by using the ImageJ program. Pixel intensity for each time point is reported in the graph (left and right panels, respectively).

Furthermore, we confirmed that MA 39/76 preserved high stability to pH changes by incubating the aptamer in buffers at different pHs (Figures 6A–C). First, we analysed the integrity of the aptamer on a denaturing gel without observing any signal of degradation (Figure 6A). Then, since the aptamer is more likely to change structure in harsh pH conditions, we performed CD spectroscopy. CD spectra allow determining the overall conformation adopted by an aptamer under given solution conditions and thus discriminating among different nucleic acid secondary structures, e.g., G-quadruplex- vs. duplex-forming oligonucleotides (Riccardi et al., 2017; Moccia et al., 2019). Specifically, we recorded the CD spectra of the aptamer from 200 to 320 nm in PBS buffer at three different pH values, i.e., at pH 5.0, 7.0, and 9.0, and at room (20°C) and physiological (37°C) temperatures. The CD spectrum of the aptamer at pH 7 and 20°C evidenced an intense positive band at 266 nm, a weak broad negative one centred at 238 nm, a narrow negative band at 211 nm, and a very small minimum at ca. 300 nm (Figure 6B, black line, and Figure 6C), all indicative features of mainly A-form RNA helix (Kerwin, 2000). The absence in the aptamer CD profile of a 280 nm shoulder evidenced the low content of single-stranded or unstructured regions (Popovic and Greenbaum, 2014), indicating that loops are mainly all structured. Notably, only small differences in the CD profile of the aptamer were revealed at pH 5 and 9 with respect to pH 7, essentially evidencing the same overall conformation adopted at neutral pH also in acidic and basic conditions (Figure 6B, red and blue lines, respectively, and Figure 6C), in line with the gel electrophoresis analysis. We also observed that the CD spectra of the aptamer recorded immediately after dilution in the selected buffer (time = 0, black line, Figure 6D for pH 5, as a representative example) and after 3 or 48 h of incubation in the buffer at 37°C (Figure 6D, red and blue lines, respectively) were almost superimposable. From these data, a clear high chemical stability upon time of the aptamer, also in extreme conditions (pH 5 and 9), emerged. Further, the spectra of the aptamer in the three different buffers at physiological temperature revealed that the aptamer was still well structured at 37°C (Supplementary Figure S1). We, thus, decided to acquire thermal stability information on the aptamer in the buffer mimicking the hypoxic condition and performed a UV-thermal denaturation experiment of the aptamer solution in the PBS buffer at pH 5. Monitoring the absorbance at 260 nm upon increasing the temperature from 20 to 90°C, we observed a melting curve with two distinct sigmoidal transitions (cooperative unfolding) at about 46 and 76°C (Figure 6E), revealing two main stems in the RNA aptamer structure. In addition, the high hyperchromic effect (26%; ΔAbs_260_ = 0.09, 20–90°C) indicated strong base-pair stacking.

In addition to pH stability, resistance to serum nucleases represents a crucial prerequisite for the clinical translation of RNA diagnostics and therapeutics. Our aptamers were modified with 2′F-Py which enhance the resistance to nuclease degradation. In order to assess the MA 39/76 serum stability, we incubated the aptamer in high human serum concentrations for different times. In order to have the most aggressive condition to evaluate aptamer stability, we used the maximum possible serum concentration (i.e., 80%), which is even higher than the physiological concentration (55%). We found that the sequence has good serum stability with an estimated half-life of about 24 h (Figure 6F). This stability allows an adequate time window for testing the aptamer *in vivo* as a diagnostic and therapeutic tool (Khalid et al., 2018; Smith et al., 2018). The result thus suggests a great applicability of the sequence for diagnostic and therapeutic purposes that is further reinforced by the versatility of the aptamer chemistry allowing the easy introduction of additional modifications to modulate their stability (Shigdar et al., 2013).

Collectively, these data confirm that the selection strategy adopted allows the isolation of aptamers with improved specificity for the hypoxic cancer cell phenotype and with high stability in acidic conditions and in human serum.

Despite the ascertained potential of hypoxia in tumorigenesis and drug resistance and the importance of its selective detection, effective hypoxia probes are lacking.

Here, we described an innovative selection strategy to target hypoxia in cancer and provided evidence of its efficacy in isolating promising nucleic acid candidates for the further development of new effective cancer diagnostics and/or therapeutics.

The standard cell-based SELEX method was properly improved by defining an innovative and simple procedure to select stable RNA aptamers against multiple hypoxia-related epitopes expressed on the tumour cell surface in their natural structure and distribution. Interestingly, such an approach may show wide applicability to different cancers or other hypoxia-associated pathologies.

Applying this method, we isolated a pool of aptamers that can specifically recognize the hypoxic phenotype and further characterised one of the selected sequences (MA 39/76), demonstrating its high affinity for the hypoxic cells (K_D_ in the nanomolar range) and its good stability to pH variation and serum degradation (half-life longer ≥ 24 h in 80% human serum). Our results thus provide a source of molecules useful for the detection of hypoxia in cancer and, more generally, in all hypoxia-associated conditions. Further, they offer promising tools to improve the efficacy of cancer therapeutics by permitting drug delivery to the hypoxic areas that are characterised by reduced drug penetrance (Shannon et al., 2003; Primeau et al., 2005). The isolated molecules may offer exquisite advantages in terms of specificity, stability, and tissue penetrance and can be easily modified to further modulate their profile for future *in vivo* applications. Although the MA 39/76 target remains to be determined, our results suggest its ability to specifically recognize a hypoxia-related cell surface epitope whose identification may allow finding out and/or validating new markers associated with the hypoxia condition.

In conclusion, given the great potential of aptamers, our study may pave the way to the development of new effective and safe modalities for hypoxia targeting, overcoming the limitations and inconveniences of currently available methods for hypoxia detection in terms of specificity and efficacy at low oxygen levels.

## Data Availability

The raw data supporting the conclusions of this article will be made available by the authors without undue reservation.
