# Supplementary material for: Selection of RNA aptamers targeting hypoxia in cancer
Source: Front Mol Biosci. 2022 Sep 14;9:956935. doi: 10.3389/fmolb.2022.956935 (PMC9515380; doi:10.3389/fmolb.2022.956935)
Supplement: Supplementary file 1 [file Table1.DOCX]

**Supplementary Table 1.** **Aptamer individual sequences.** Central random regions are reported.

| **MA Aptamer** | **Sequence (5’-3’)** |
| --- | --- |
| 1 | GUUUCCGGAUUGCUUGCCUUGCUGUCGUUUCCAACCUCUC |
| 2/21 | UUCUGCUUCCAUGUUUCCACCGUGAUUCCGGGUUUCGCCG |
| 3 | UCCGGUAAACCUUCACGAUAUUUUCUUUUCUAUCGGCAUG |
| 4 | AUGACGUUAGCAUCGUGCGGAGUGGUGGUCGCCCUCGAUG |
| 5 | CAUAAUGAGUACUGAGUUAUAAGCUGGGAAUACUGUGCGC |
| 6 | GAAUUGUGAUAGAGUCGCGAAUGAAGCAAGUAGUUUGCAG |
| 7/15 | CCUUAUUCUCUUGCGAAUACUGAGGGCCUGACGUUUCCCA |
| 8 | UGUGUUGUCGUCUCGCUGGAGUUCGUCUGCUUCCGACGCA |
| 9/70 | UCGUAAUGUGUAUCGUAUGGCUUAUGACAAGUCCGUAAUG |
| 10 | ACAGUACUGCGUAAAGUUGGGGGAUACACGCGCGGUACC |
| 11 | CAGCACACAAGGUUGUCGAAUAGGAGUGGUGCCUAACCC |
| 12 | CCGAAGGUAAGUCACGCCAUGAUUGAAAGUUUGUGCCCC |
| 13/40 | UGAGAAUGUACUGUAGCCAGUUCGACUACUGGAUACCUCC |
| 14 | GCACGUUUGAACAGCUACUGUGUGGUGGUCGUAUCCACAG |
| 16/18 | UCCUCACAGUGUGUAACCCUACCUUUCUUCAUAGCCACGG |
| 17 | GCGAUGUAGUGGCGUCUCUGCAGUUUUGCUGCAAUGACUG |
| 19 | CGCAGUAGUCUAUUUGGUGCACUGUGCGCUGUCAGUGCUG |
| 20 | AAACAGCAACGCGGGGCAGUGGACGGAUACAAUGCAGUCC |
| 22 | AGAGUGCUCAAACGGCGAUGCAACAUCGGUUUGGUAUCCG |
| 23 | CAGCGUCAUGCGCGGUGCCGUCACGCGCUACUAGUUUUGG |
| 25 | CGUAUGAAACAUAGUACAGGCAUUGCGCAGGCGCCGCGAC |
| 26 | GGUAUGAGAGAUACACCAGUAGAUUCGGCUGGUUCGGUCG |
| 27 | GUUACGUGCUGAUGAGGCAGGCGCGAUUUAAGUGCUUAUG |
| 28/100 | AAAUCAUGUACGUCACUGUGUCGGUGUCGAGGGACCCGUG |
| 29 | ACCGCCGUCAUGGCCGUGGCUUGUAGGUGCAAAUAGCGUG |
| 30 | AACGGGAGUACGAUUCGCAGCACAGGGCUCGGUCAAUG |
| 31 | ACUGCUUCUAUUUCUUUUGUCGCCAAUUCACCAUCUCGA |
| 32 | CUUUGUGGGACACAGCCACAGCAAGGUGUGCUGUUGCGUG |
| 33 | ACUGCGGGCACAGAUAGCGAACGAACCGAUCACUCAGGCG |
| 34 | UUAAAAUUUCUCCCUCAAGGUCCUCCAUUCGGUCUCCUGG |
| 35 | CUACUUUCAAACAAGUGCUUUUUCGAAACCUUGACACCGA |
| 36 | GCAAAACGAUUAGGUGAGUGCCCCCAUACCUGAUGCGCUC |
| 37 | CACGUGCGGGGUAAGCAUUCACGUGAUUCACAAUUGUG |
| 38 | CGACAAGUGAGUUGCAUAACGCUCGAGGGGAAGCGUAUGA |
| 39/76 | CGCUGUGUGGAUAACAUGUAGCUACACAAUUCGAACCGC |
| 41 | UCUGCUUCCAUCGAAUAAUCACUCCGUUACAGUGCGUCGC |
| 42 | CGAGGUGAUGUAAUAGGGCACACAUUGGUUUGGUCAUCAG |
| 43 | CCUGCACAUAUGGUUACCUCUUACAGUACUCCUUCUCACA |
| 44/90 | UCGCCGUAUUCUCCUUAGAUCGUAUAGAUUUAAGGGUUGA |
| 45 | CGGGUCACACAACGUAGUACUAGUGCCCCCAAACUGCGCG |
| 46 | GCAUGUUUCGCCAUUGGACGACAUGCUUGAUCUCAGGCA |
| 47 | CGCGCACAAUGGUAAUGGGUGGUUUGCGCGUGUGGUUCUG |
| 49 | CCGUAUGAGGAAGCCGCGGCAGGCUCCAUGACACGUCUGG |
| 50/96 | ACCGCCGUAUGCGUAGCAUCCAUUGUAUAACGGCGACGCC |
| 51 | CCGACACCUUUCGCCUAGGCUAACGUCACCUUGUGUCAUG |
| 52/58 | GACGUGUGGUAAACUGCGAAGUUUCGGGGAUCCGCAGUUG |
| 53 | CCAUGUAAAGACUGUAUAACGGUUACUGCGUCAGUCUUGG |
| 54 | CCAUGUAAAGACUGUAUAACGGUUACUGCGUCAGUCUUGG |
| 55 | GCGUUGUGGUUUGAUUUGGGUUCGUUCGCCAAUUCGGUAA |
| 56 | UACAAGCGAGUGCGGGUCUAACCGAUAUGUGGUCCUCGC |
| 57 | CCAUUUGCUCAUCUACUACUCAACUUUUUUGCCUGCGACG |
| 59 | UCCUUUACUGAUGCGUUCCCUAACACCUCGUUCGCACGG |
| 60 | CGCUUAUUAGUUUUCUCACACAUCUUCCACUCUUCAGGCG |
| 61 | CCAACGUAUGUCGUCACACGCGCGCAUACGCUAUACCUGG |
| 63 | UCGGGUCGGCAACUAGUGGUGUGCACGCGCUACGGUAUUC |
| 64 | CCGGUAUGGCAGGUAGAGCCCUUAGUGGGGGGCUUUGGUG |
| 65 | CCAUACAGCUGCGCAACGCCAAUAAGGAAACUCUCGAUGG |
| 66/97 | UCUUUCGCGAAGUCUCGUCAGCUGCGUAAACAAAACUCCC |
| 67 | GUGACUUCAUUUCGCCAUCCUUUCUCUAAACGGCAACCGA |
| 68 | CGAGUACCCGUCUUAUUGAACUGCGGGCAACUGUUGGUGG |
| 69 | UCGGGUUAUCGGGUCGCUGUUAUCGUUUCCUUCGAC |
| 71 | UCUUCAUACAUUCACGUGCGCGGUGAGUGUUUUGGCGUGU |
| 72 | CUCGCUUUGCACCGUUUACACUCCCCUUCUCCCCCUGUCG |
| 74 | UCUUUGUCACGCUGCGAUACACACCUUACUGGUCUCUUCC |
| 75 | CACAUGCCACAUGCGGUUGCUAGUUAACGUUUGCCCGCAG |
| 77 | AACUUGGCCCAUUUCAUACGUCUUUUAACUGGUGGCUUGG |
| 78 | CGGCAAAUUUCUGUUUAUCCGGGCGCACAAGUGCUCUUGA |
| 79 | GCGUAUCGUGUAAGGGCAAACGGGCUGCCCUUCGUCGUG |
| 80 | AUUUGGUGGCUUAGUGAUUGAGGUGCAUCUUAAGUGAGCA |
| 81 | GGCUCGGAUAACACCGGCAAAGAUCGAGCAUCUGUCAUGG |
| 82/98 | UGGAACAUUGGAAGUCAGAACGGCUAAAGGUGAUAGGUUC |
| 83 | UCCGACCAGUGAGAGUAGUGGGUGGUUCUCACUGCGCGU |
| 85 | UGUGCUGCAACGAACAGUGGAUUGGGCAAGGCGAUGCUCG |
| 86 | GCUCAACUGGUAGUGUCUCUCAAUGGGCCGAAUUGCACUC |
| 87 | CGCGGGUCACAAGUGUGGUAGAGCACUGUGCUGUACGGUG |
| 88 | ACCUUUCCACGUUUGGUUACGUGGGUUACUAGUUUUCGCC |
| 89 | CAUAUGUGGAACGGGUUGAACUUGGAUGUGCACCUUUUGG |
| 91 | CCUUAGCGAUGAGAUUGGAGGUUGCUCGUCGCUCGUUUGG |
| 92 | ACAUCACGCUUUGCGAUCGGUAUUGGGGAUAAGUGCCGUG |
| 93 | CUUUGUGGAGUGCGUCCAUUGCAGAACCAAUGUGGGCUGA |
| 94 | CGCAUGACAUCGCGUUAAACAUUGAUGUGUCGCGUACUG |
| 95 | CGAUUGGUGAGGGCGUCGUCAAUCGAACUCGCUGGUUAAC |
| 99 | CCACUCUUGGCGUAUUGUGAUCGGUUGAAUGUGGCAUCG |
