# Supplementary figures and images for: Selection of RNA aptamers targeting hypoxia in cancer
Source: Front Mol Biosci. 2022 Sep 14;9:956935. doi: 10.3389/fmolb.2022.956935 (PMC9515380; doi:10.3389/fmolb.2022.956935)

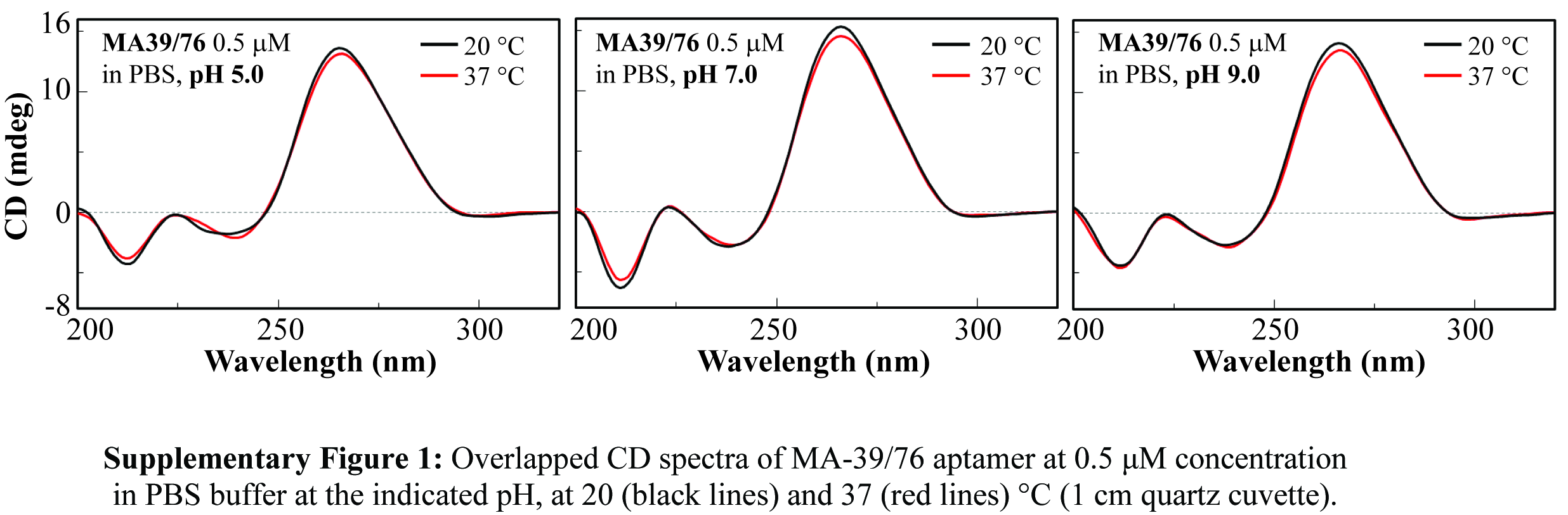

Supplement: Supplementary file 2 [file Image1.TIF]
